# Maternal Cardiac Function after Normal Delivery, Preeclampsia, and Eclampsia: A Prospective Study

**DOI:** 10.1155/2019/9795765

**Published:** 2019-03-03

**Authors:** Elena Timokhina, Tatiana Kuzmina, Alexander Strizhakov, Elena Pitskhelauri, Irina Ignatko, Vera Belousova

**Affiliations:** I.M. Sechenov First Moscow State Medical University, Department of Obstetrics, Gynecology and Perinatology, Moscow, Russia

## Abstract

**Introduction:**

The aim of this study is to assess maternal cardiac function in the postpartum period, after 2 and 6 months in the parturient with preeclampsia and eclampsia.

**Materials and Methods:**

Prospective study: 90 postpartum women after preeclampsia and eclampsia and 55 patients after an uncomplicated pregnancy. The parameters of maternal hemodynamics were recorded on days 1, 3, 5, 9, and 14 of postpartum period, after 2 and 6 months. The cardiac parameters were assessed.

**Results:**

PE is accompanied by increased peripheral vascular resistance. The indicator of vascular resistance, SVR, is elevated for both mild and severe PE. With mild PE, a significant increase in SVR is observed up to 5 days of postpartum period, with severe PE/E up to 9 days. We found that in case of severe PE, SVR remains elevated to 6 months after delivery. The parameters of the contractile function of the heart (ESV, EDV, SV, SI, CO, СI, MVCF) were significantly decreased: with mild PE up to 5-9 days, with severe up to 9-14 days of puerperia. ESV, SV, SI, CO, and CI remain low with severe PE up to 6 months. The revealed decreasing of contractile function of the heart is a sign of asymptomatic heart failure.

**Conclusions:**

The hemodynamics of the puerperas after PE and E is characterized by impaired contractility of the myocardium and an increase in the indices of peripheral resistance. The degree of deviation in the parameters of cardiac hemodynamics and vascular resistance depended on the severity of hypertensive complications of pregnancy.

## 1. Introduction

Understanding the main features of maternal hemodynamics in normal pregnancy as well as in various obstetric pathologies is the basis for prediction of pregnancy complications, perinatal outcomes and choosing the right treatment of pregnancy disorders.

Hypertensive disorders during pregnancy are present in about 10% of pregnant women, with preeclampsia being seen in 2-8% of all pregnancies [1, 2].

Every year more than 50,000 women worldwide die during pregnancy due to complications associated with hypertension. In developed countries, arterial hypertension and preeclampsia are the second direct cause of ante- and postnatal mortality due to preterm delivery [2].

Hypertensive disorders take the 4th place in the list of causes of maternal mortality in the last decade [1]. Moreover, they cause severe maternal and fetal morbidity and disability.

However, with proper management majority of adverse outcomes are preventable. Severe hypertensive disorders during pregnancy can also reduce postpartum quality of life for women often leading to increased frequency of chronic hypertension, diabetes mellitus, myocardial infarction, and stroke, as well as increase frequency of impaired physical and psychosomatic development of premature infants which in turn can lead to significant social and health issues [3–19].

The evidence is now clear that preeclampsia is associated with higher risk of developing cardiovascular disease later in life [4, 5, 11, 16, 18, 19]. However, further research is needed to determine how best to use this information to help patients.

The objective of this study is to assess maternal cardiac function immediately in the postpartum period, and also after 2 and 6 months, in the parturients who were diagnosed with preeclampsia and eclampsia.

The part of the study was presented as a poster at 25th World Congress on Controversies in Obstetrics, Gynecology & Infertility (COGI) Vienna, Austria, November 30–December 2nd, 2017.

## 2. Materials and Methods

This is a prospective observational case-control study done between 2012 and 2015. The study included only women with singleton pregnancies.

Preeclampsia was defined as the new onset of hypertension and proteinuria in human pregnancy after the 20th week of gestation [2].

*Diagnostic Criteria for Preeclampsia*. Preeclampsia is diagnosed when systolic blood pressure is greater than or equal to 140 mm Hg or diastolic blood pressure is greater than or equal to 90 mm Hg on two occasions at least 4 hours apart after 20 weeks of gestation in a woman with a previously normal blood pressure and proteinuria with excretion of 300 mg or more of protein per 24-hour urine collection (or this amount extrapolated from a timed collection) or protein/creatinine ratio greater than or equal to 0.3 (each measured as mg/dl) (Dipstick reading of 1+ can be used only if other quantitative methods are not available). We consider all patients meeting criteria of preeclampsia have mild preeclampsia (without severe features). If any features of severe preeclampsia are registered at any gestational age, we consider these patients to have severe preeclampsia.

*Severe Features of Preeclampsia (Any of These Findings)*

- Systolic blood pressure of 160 mm Hg or higher, or diastolic blood pressure of 110 mm Hg or higher on two occasions at least 4 hours apart while the patient is on bed rest (unless antihypertensive therapy is initiated before this time)
- Thrombocytopenia (platelet count less than 100,000/microliter)
- Impaired liver function as indicated by abnormally elevated blood concentrations of liver enzymes (to twice normal concentration), severe persistent right upper quadrant or epigastric pain unresponsive to medication and not accounted for by alternative diagnoses, or both
- Progressive renal insufficiency (serum creatinine concentration greater than 1.1 mg/dL or a doubling of the serum creatinine concentration in the absence of other renal disease)
- Pulmonary edema
- New-onset cerebral or visual disturbances

All patients with mild and severe preeclampsia in the postpartum period were under intensive care for 2-4 days, where they were treated with a complex therapy based on current international guidelines for the treatment of preeclampsia and eclampsia.

The survey was conducted on days 1, 3, 5, 9, and 14 of postpartum period, as well as after 2 and 6 months after delivery. We included only those cases that could be traced throughout the entire study period after delivery.

We measured cardiovascular parameters noninvasively by echocardiography.

Exclusion criteria included chronic diseases of the cardiovascular system and kidneys. Women with medical comorbidities, smokers, on medication, or with fetal abnormalities were excluded from recruitment in the study.

The study was conducted using an ultrasound diagnostic system Accuvix-A30 Samsung Medison company. Phased probe 2-4 MHz was used to perform echocardiography.

The following indicators were assessed by echocardiography: end-systolic volume (ESV), end-diastolic (EDV), stroke volume (SV), cardiac output (CO), stroke index (SI), cardiac index (CI), mean velocity of circumferential fiber shortening (MVCF), heart rate (HR), mean blood pressure (MBP), and systemic vascular resistance (SVR).

Echocardiography was performed by one investigator. All conventional echocardiographic indices were adjusted for body surface area.

### 2.1. Statistical Analysis

Data sets were analyzed and are presented as means ± SD. For comparison of data, sets were tested for normal distribution (Shapiro-Wilk test). To compare outcome parameters between groups of healthy and pathologic pregnancies, an analysis of variance was performed. The Pearson correlation coefficient was calculated to analyze relationships. Time points were compared pair-wise with a Sidak correction for the significance level due to the multiple comparisons applied herein.

P values (two-sided) lower than 0.05 were considered statistically significant. Statistical analysis was performed with SPSS 21 (SPSS Inc., Chicago, Illinois, USA).

## 3. Ethical Approval

All women provided informed consent and the study was approved by the local review committee (Reference number 5-12).

## 4. Results and Discussion

After providing written informed concerns, a total of 145 women were included in this study.

In order to study the maternal cardiac function after labor, we examined 55 patients after an uncomplicated pregnancy (control group) and 90 postpartum women with mild and severe preeclampsia and eclampsia (main group) at any gestational age.

Demographic and pregnancy characteristics in the cohorts of patients are presented in Table 1.

The maternal age of healthy women of control group was 24,8 ±3,08 year. Pregnancy in all patients was uneventful and ended with term delivery at 38-40 weeks. The average gestation period at the time of birth was 39,4± 0,75 weeks.

Cesarean section deliveries were performed for indications not related to hypertensive disorders for all patients in the control group.

Blood loss after caesarean section was 610 ± 75.8 ml. All infants had Apgar scores of 8-9 points.

The average weight of children at birth was 3445 ± 405,8 with individual variations from 2800 g to 4400 g; newborn height was 51,43±2,75 cm, with individual variations from 49 cm to 56 cm.

The average age of patients in the main group was 26,7 ± 1,6 years. Nulliparous women represented 61 (67.8%), multiparous 29 (32.2%). It should be noted that 20 (68.9%) of the 29 multiparous women had previous pregnancy complicated by preeclampsia.

In the analysis of somatic anamnesis of women in the main group, it was found that 22 (24.5%)of patients had extragenital pathology. Diseases of the digestive system were observed in 17 (18.9%) postpartum women with preeclampsia, infections of the upper respiratory tract in 31 (34.4%), overweight in 7 (7.8%), and eye diseases 13 (14.4%).

There were no significant differences in somatic anamnesis among control, the main group.

43.3% (39) of the women were found to have mild preeclampsia, 52.2% (47) severe preeclampsia, and 4.4% (4) eclampsia. It should be noted that during pregnancy eclampsia occurred in 3 (3.3%) of women, and during the first 48 hours of postpartum period in 1 patient.

In the main group, gestational age at the time of delivery ranges from 26 to 39 weeks (Table 1). Early onset preeclampsia was revealed in 51,1% (46), late-onset preeclampsia 48,9%(44).

All patients had to be delivered by caesarean section due to severe preeclampsia, or combination of mild preeclampsia and progressive fetal distress.

Blood loss after caesarean section in main group was 622 ± 63.3 ml.

In the main group a total of 88 children were born with 2 fetus dead prenatally and 3 infants who died in the early neonatal period. Thus, the overall perinatal loss was 5 -5.6%.

It should be noted that all cases of perinatal death were seen with severe preeclampsia and eclampsia and accompanied by severe fetal growth retardation (below the 10th percentile) and very preterm cesarean labor. The average weight of the newborn, including stillbirths, was in the group with a mild preeclampsia 2747,6± 320,6 g, with a severe - 1912,2 ±236,2 g (p <0,01).

### 4.1. Maternal Hemodynamics of Healthy Postpartum Women

Parameters of cardiac hemodynamics in women after an uncomplicated pregnancy as well as patients with mild and severe preeclampsia are presented in Table 2.

Comparing the volume of central hemodynamic parameters showed a significant (p <0,05) increase in EDV on the 3rd day after the birth of 11.9% with no significant change (p<0,05) of ESV. At the same time, there is an increase in the SV and CO by 21.3% and 24.7%, respectively.

**Figure 1:**
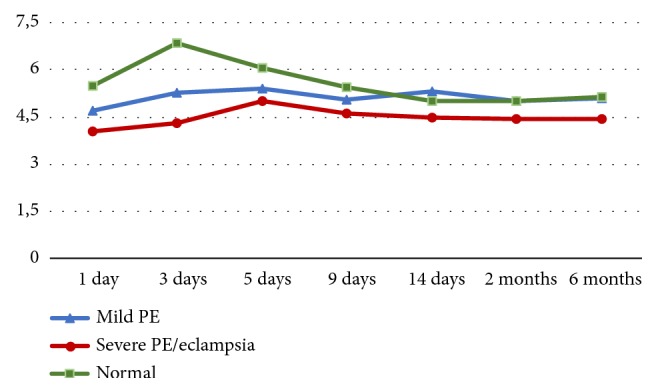
*Parameters of cardiac output (CO, l / min) in noncomplicated patients, after preeclampsia and eclampsia*. The values of cardiac output (CO) were significantly decreased in the postpartum period in the patients of the main group (p <0.05): with mild PE up to 5-9 days and severe up to 9-14 days of puerperia. Cardiac output remains low with severe PE up to 6 months (p> 0.05).

Due to dependence of indicators of cardiac hemodynamics on constitutional features of the subjects, as well as decrease in woman's weight after labor, we calculated CI (Figure 1) and SI.

Conversion of SV and CO per unit body surface area also confirmed that on the 3rd day of postpartum period occurs a significant (p <0,05) increase in SI and CI by 18.9% and 21.6%, respectively. In this case the maximum values of SI are 46,34 ±2,01 ml/m^2^, CI 3,72± 0,19 l/min/m2.

Subsequently, the average values of ESV, EDV, SI, and CI gradually declined. We did not observe any changes in the cardiac parameters after 2 and 6 months postpartum. The average values of SVR declined on 3rd day after delivery by 11.6% and then increased by the end of the second week postpartum period by 26.1%.

Thus, after turning off the uteroplacental blood flow and reducing blood volume in the circulatory system, puerperal patients develop compensatory-adaptive reactions, which are characterized by an increase of cardiac indicators of maternal hemodynamics and reducing systemic vascular resistance.

### 4.2. Maternal Hemodynamics of Postpartum Women after Preeclampsia-Eclampsia

In order to identify the typical changes in cardiac hemodynamics after mild and severe preeclampsia we examined 90 patients on days 1, 3, 5, 9, and 14, 2 and 6 months after delivery.

At 43.3% (39) of the women were diagnosed mild PE, 52.2% (47) severe PE, 4.4% (4) eclampsia.

Our studies have shown that women with mild and severe of preeclampsia have disorders of the cardiac function, manifested in significantly lower values of volume indicators and the contractility of the myocardium with increase in mean blood pressure and systemic vascular resistance.

PE is accompanied by increased peripheral vascular resistance. According to our data, the indicator of vascular resistance, SVR, is elevated for both mild and severe PE.

With mild PE, a significant increase in SVR is observed up to 5 days of postpartum period, with severe PE/E up to 9 days. The absolute values of SVR with mild PE were 1422.31 ± 117, 08 dyn • s • cm-5, with severe PE 1702.31 ± 127.08 dyn • s • cm-5 (in healthy puerperas -1200.3±103,23 dyn • s • cm-5).

We found that in case of severe PE, SVR remains elevated to 6 months after delivery (p> 0.05).

The parameters of the contractile function of the heart (ESV, EDV, SV, SI, CO, СI, MVCF) were significantly decreased in the postpartum period in the patients of the main group (p <0.05): with mild PE up to 5-9 days and with severe up to 9-14 days of puerperia (Figure 1).

ESV, SV, SI, CO, and CI remain low with severe PE up to 6 months (p> 0.05).

The revealed decreasing of contractile function of the heart is a sign of asymptomatic heart failure.

## 5. Discussion

Our data shows that maternal hemodynamics in the puerperium in healthy women and patients with preeclampsia are significantly different.

The results of our echocardiographic study of healthy postpartum women that were delivered by cesarean section according to indications not related to hypertensive disorders reveal a significant increasing of EDV, SV, and SI on day 3 after delivery by 18.9%, 9%, and 21.6% respectively and decreasing of SVR, compared with the same indicators in the first day postpartum.

In addition, there is an increase in the rate of MVCF, indicating an adequate cardiac pumping function in the setting of increased EDV.

Further, due to the decrease in the volume of circulating blood and the total vascular volume, we demonstrated gradual decrease in the main cardiac indices and an increase in the SVR.

This is consistent with earlier studies [19], where a gradual decrease in cardiac output in the puerperium was observed due to a decrease in heart rate and stroke volume.

In the study of hemodynamic profiles in patients with mild and severe forms of preeclampsia, we demonstrate significantly (P <0.05) lower values of EDV, SV, and SI and elevated SVR values in the first 24 hours after delivery.

We demonstrated that a decrease in volume indices of cardiac function correlates with the severity of preeclampsia. In addition, after severe preeclampsia, increased ESV values and reduced MVCF parameters indicate decrease in the contractility of the myocardium.

This does not conflict with the data of several authors [5, 7, 9–11, 13, 17] that preterm PE disorders of hemodynamics lead to seriously impaired and long term cardiac dysfunctions.

The important study was conducted by T. Ghi et al. [12]: echocardiography including tissue and pulsed Doppler revealed that among women who experienced a pregnancy complicated by severe preeclampsia the incidence of asymptomatic ventricular dysfunction at postpartum echocardiography is significantly increased. LV contractility and diastolic function, although within normal reference ranges, show slight but significant impairment. According to authors, a follow-up echocardiography at short distance (6–12 months) from a preeclamptic pregnancy may represent a mandatory challenge test which may adjust the risk of subsequent cardiovascular events in accordance with ventricular function findings.

Ghossein-Doha C. et al. [16] found that the prevalence of asymptomatic HF-B long term after delivery was approximately 3.5 times higher in the PE group than in the control group. Earlier the same conclusions were made by Melchiorre K., Thilaganathan B. et al. [13, 14].

We demonstrated that disorders of hemodynamics in the puerperium are observed in almost all patients with mild to severe preeclampsia and manifested by dissociation between the SV and SVR, indicating reduced compensation of circulatory system.

According to our data, recovery of hemodynamic parameters in the postpartum period directly depends on the severity of preeclampsia.

Orabona R. et al. [17] also demonstrated that women who underwent EO-PE are more likely to have subclinical disruption of systolic biventricular function than those who had a history of LO-PE or who did not have hypertensive disorders of pregnancy.

Murphy MS. et al. [15] found that after preeclampsia the heart rate remained low for a long time, indicating a conduction disorder and an increased risk of arrhythmia in such patients.

Tihtonen K. et al. [20] also revealed dysfunctional changes in left ventricular myocardium in patients with preeclampsia, which leads to low CI and SI values.

Bellamy L. et al. [18] demonstrated that in women after severe preeclampsia the risk of CVD is increased (hypertension, coronary heart disease, stroke, and thromboembolic conditions).

These data are consistent with our findings that patients with preeclampsia are at high risk of the development of cardiovascular complications in the long term.

## 6. Conclusions

Hemodynamics of women whose pregnancies were complicated by PE/E has significant differences from hemodynamics of postpartum women that were delivered by cesarean section due to indications not related to hypertensive disorders. The major issue is impaired contractility of the myocardium and increased values of peripheral vascular resistance. The degree of deviation in the parameters of cardiac hemodynamics and vascular resistance depends on the severity of hypertensive disorder of pregnancy.

Thus, patients after PE/E are at high risk of long-term cardiovascular disease, requiring cardiologic follow-up and tight control of blood pressure.

When planning a subsequent pregnancy, we recommend assessing cardiac function, tight blood pressure control, and administration of antihypertensive therapy to achieve it.

## Figures and Tables

**Table 1:** Baseline characteristics of study cohort.

Parameter	Mild PE (n =39 )	Severe PE (n =47)	Eclampsia (n=4)	Normal pregnancy and labour (n =55)
Maternal age (years)	31.5 (26–34)	31.8 (26–35)	33.4 (30–37)	24,8 (20-32)

Prepregnancy BMI (kg/m2)	22.6 (20.8–25.0)	23.2 (21.6–27.3)	25.8 (22–31.5)	22.5 (20.8–25.8)

Gestational age at delivery (weeks)	35,6 (32.2–39.1)	33.1 (26.5–38.7)	34.3 (31.6–37.3)	39.4 (37,8–40,4)

Weight of newborn	2747(1500-3200)	1912(800-2970)	2250 (1800-2570)	3445(2800-4400)

HR (beats/min)	81 (75–87)	79 (71-85)	85 (72–89)	79 (74–92)

**Table 2:** Hemodynamic parameters of postpartum women with uneventful pregnancy, mild preeclampsia, and severe preeclampsia/eclampsia* (M ±SD).*

**Hemodynamic parameters**		**Period after labor**						
		**1 day**	**3 days**	**5 days**	**9 days**	**14 days**	**2 months**	**6 months**

**Systolic blood pressure**, mm Hg	***Mild PE***	139,41±3,01*∗*	124,42±4,24*∗*	119,24±4,47*∗*	109,62±2,17	108,86± 4,45	106,38± 4,38	107,33± 4,45
	***(n=39)***							

	***Severe PE/eclampsia***	158,51±4,21*∗*	145,83±4,51*∗*	131,44±3,47*∗*	123,84±3,11*∗*	111,86±3,45	106,58±3,15	108,46±3,43
	*** (n=47+4)***							

	***Normal***	103,31±3,21	104,23±4,74	103,94±4,51	102,42±3,67	102,46± 4,45	103,34± 4,45	103,71± 4,11
	***(n=55)***							

**Diastolic blood pressure**, mmHg	***Mild PE***	93,06± 2,13*∗*	87,96± 2,33*∗*	82,36± 3,07*∗*	79,6± 2,38*∗*	75,88± 3,43	73,81± 3,23	74,31± 3,14
	***(n=39)***							

	***Severe PE/eclampsia***	106,56± 2,13*∗*	100,26± 3,35*∗*	92,16± 2,57*∗*	92,81±2,68*∗*	79,84±3,85	72,81±3,53	73,66±3,61
	***(n=47+4)***							

	***Normal***	69,24± 2,43	72,36± 3,12	71,16± 2,07	71,7±2,81	70,24± 4,09	69,41± 3,21	68,82± 3,28
	***(n=55)***							

**MBP**, mmHg	***Mild PE***	108,31± 3,12*∗*	100,88±3,71*∗*	94,62± 2,09*∗*	89,71± 2,04	86,85± 3,04	84,64± 3,03	84,92± 3,13
	***(n=39)***							

	***Severe PE/eclampsia***	123,81± 3,82*∗*	115,48± 3,21*∗*	105,28± 3,09*∗*	103,14±4,04*∗*	89,88±3,04	83,40±4,03	84,81±3,58
	***(n=47+4)***							

	***Normal***	80,59± 3,14	82,98± 4,67	82,09± 3,16	81,94± 4,78	80,98± 3,01	80,72± 3,93	80,12± 3,14
	***(n=55)***							

**HR**, beats / min	***Mild PE***	89,57± 2,31*∗*	87,52± 2,11*∗∗*	84,16± 2,14*∗*	78,27± 1,18	77,32± 1,19	77,03± 2,28	76,12± 1,88
	***(n=39)***							

	***Severe PE/eclampsia***	96,56± 2,11*∗*	91,82± 2,12*∗*	89,19± 2,19*∗*	79,17± 2,19	74,37±1,13	71,12±2,18	72,32±3,11
	***(n=47+4)***							

	***Normal***	78,41± 2,33	80,34±2,26	76,6±1,54	76,4±2,13	75,34±1,92	75,12± 2,13	74,14± 2,03
	***(n=55)***							

The end-systolic volume of the left ventricle (**ESV**), ml	***Mild PE***	48,32± 2,14	49,04± 3,26*∗*	46,08± 2,21	44,12± 2,11*∗*	41,96± 2,12	40,41± 3,42	40,18± 3,51
	***(n=39)***							

	***Severe PE/eclampsia***	51,61± 2,62*∗*	55,06± 2,05*∗*	49,26± 2,81*∗*	47,24± 2,42*∗*	42,76± 2,12*∗*	43,62±3,12	44,21±3,04
	***(n=47+4)***							

	***Normal***	42,50± 2,45	40,41± 2,21	40,81± 2,92	38,67± 1,52	37,58± 1,32	38,80± 2,92	39,07± 2,17
	***(n=55)***							

The end-diastolic volume of the left ventricle (**EDV**), ml	***Mild PE***	101,19± 3,01*∗*	109,06± 2,21*∗*	109,32± 2,19*∗*	109,14±2,85	106,14± 2,45	105,51± 2,35	104,66± 2,48
	***(n=39)***							

	***Severe PE/eclampsia***	93,26± 2,41*∗*	102,07± 2,21*∗*	105,38± 2,08*∗*	105,36±2,83	105,01± 3,15	105,52±2,15	104,23±2,69
	***(n=47+4)***							

	***Normal***	112,61± 2,98	125,49± 2,01	120,18± 2,01	109,72± 2,15	105,20± 2,01	105,63± 3,94	104,72± 3,25
	***(n=55)***							

Stroke volume (**SV**), ml	***Mild PE***	52,57± 2,01*∗*	60,62± 2,01*∗*	64,24± 2,61*∗*	65,02±2,11*∗*	69,18± 2,03	65,18± 2,61	66,29± 2,34
	***(n=39)***							

	***Severe PE/eclampsia***	41,55± 2,63*∗*	47,04± 2,45*∗*	56,16± 2,72*∗*	58,12±2,61*∗*	60,24±2,01*∗*	62,16±2,41	63,18±2,13
	***(n=47+4)***							

	***Normal***	70,13± 2,51	85,09± 2,21	79,32± 2,19	71,04± 2,01	67,62± 2,23	66,83± 2,92	65,94± 2,44
	***(n=55)***							

Stroke Index (**SI**), ml / m2	***Mild PE***	28,41± 1,61*∗*	33,86± 1,64*∗*	36,09± 2,12*∗*	37,08± 2,03	39,43± 2,82	37,88± 3,43	38,76± 3,12
	***(n=39)***							

	***Severe PE/eclampsia***	24,51± 1,31*∗*	28,24± 1,16*∗*	33,55± 2,16*∗*	34,02± 2,14*∗*	35,13±1,42*∗*	35,01±2,14	35,31±1,78
	***(n=47+4)***							

	***Normal***	38,96± 2,21	46,34± 2,01	43,51± 1,16	40,58± 1,12	39,12± 1,22	38,24± 3,13	38,11± 2,14
	***(n=55)***							

Cardiac output (**CO**), l / min	***Mild PE***	4,71± 0,22*∗*	5,28± 0,51*∗*	5,40± 0,58	5,06± 0,18	5,31± 0,56	5,02± 0,58	5,09± 0,44
	***(n=39)***							

	***Severe PE/eclampsia***	4,06± 0,31*∗*	4,31± 0,41*∗*	5,01± 0,38*∗*	4,60± 0,18*∗*	4,48±0,15*∗*	4,42±0,38	4,44±0,29
	***(n=47+4)***							

	***Normal***	5,51± 0,28	6,87± 0,21	6,08± 0,22	5,44± 0,18	5,01± 0,16	5,02± 0,31	5,13± 0,28
	***(n=55)***							

Cardiac index (**CI**), l / min / m2	***Mild PE***	2,55± 0,25	2,98± 0,39	3,10± 0,31	2,87± 0,16	2,99± 0,52	2,76± 0,55	2,80± 0,58
	***(n=39)***							

	***Severe PE/eclampsia***	2,38± 0,21*∗*	2,62± 0,19*∗*	2,96± 0,22*∗*	2,88± 0,19	2,65± 0,16	2,52±0,25	2,51±0,21
	***(n=47+4)***							

	***Normal***	3,06± 0,21	3,72± 0,19	3,57± 0,21	3,12± 0,24	2,89± 0,19	2,78± 0,25	2,71± 0,29
	***(n=55)***							

**MVCF**, s^−1^	***Mild PE***	1,20± 0,05	1,24± 0,04	1,24± 0,05	1,25± 0,05	1,26± 0,06	1,26± 0,06	1,27± 0,14
	***(n=39)***							

	***Severe PE/eclampsia***	1,12± 0,03*∗*	1,14± 0,03*∗*	1,16± 0,04*∗*	1,17± 0,03	1,17±0,03	1,22±0,08	1,23±0,11
	***(n=47+4)***							

	***Normal***	1,21± 0,04	1,25± 0,04	1,22± 0,04	1,22± 0,05	1,25± 0,03	1,26± 0,06	1,26± 0,09
	***(n=55)***							

**SVR**, dyn • s • cm^−5^	***Mild PE***	1840,03±113,32*∗*	1502,08±103,12*∗*	1426,39±102,72*∗*	1422,31±117,08	1318,82±128,16	1352,32± 122,42	1364,24± 124,16
	***(n=39)***							

	***Severe PE/eclampsia***	2444,31±123,62*∗*	2148,02±118,11*∗*	1850,59±116,82*∗*	1702,31±127,08*∗*	1412,61±131,11	1412,26± 96,02	1410,37± 102,04
	***(n=47+4)***							

	***Normal***	1170,41±92,12	1035,26±102,14	1039,0±98,21	1200,3±103,23	1310,0±105,13	1375,2± 119,01	1384,3± 112,22
	***(n=55)***							

*∗*- p<0,05– significance of differences compared with the same parameters after normal pregnancy.

## Data Availability

The data used to support the findings of this study are available from the corresponding author upon request.

## Abbreviations

CI: Cardiac index
CO: Cardiac output
EDV: End-diastolic volume
ESV: End-systolic volume
HR: Heart rate
MBP: Mean blood pressure
MVCF: Mean velocity of circumferential fiber shortening
SI: Stroke index
SV: Stroke volume
SVR: Systemic vascular resistance.

## Additional Points

*Key Message*. Postpartum period of women whose pregnancies were complicated by PE/E differs in impaired contractility of the myocardium and increased values of peripheral vascular resistance. After preeclampsia/eclampsia, echocardiography should be performed for an objective evaluation of the contractile heart function and peripheral vascular resistance.
